# Adherence to Compression Stockings for Venous Leg Ulcer Prevention: A Pilot Randomised Controlled Trial and Health Economic Analysis, Evaluating a New Multidimensional Tool (PAMCAI)

**DOI:** 10.1111/iwj.70244

**Published:** 2025-04-15

**Authors:** Laila Bar, Susan Brandis, Rachel Wenke, Darryn Marks

**Affiliations:** ^1^ Faculty of Health Science and Medicine Bond University Gold Coast Queensland Australia; ^2^ Gold Coast Hospital and Health Service Gold Coast Queensland Australia; ^3^ School of Health Sciences and Social Work Griffith University Gold Coast Queensland Australia

**Keywords:** compression stockings, cost utility analysis, PAMCAI, patient adherence, venous leg ulcers

## Abstract

Venous leg ulcer recurrence can be prevented with daily compression stocking wear; however, stocking effectiveness is often hindered by poor patient adherence. The ‘Personalised and Multi‐dimensional Compression Assessment and Intervention’ (PAMCAI) is a multidimensional decision aid, delivered by clinicians via an iPad application, designed to improve adherence. This study piloted the methodology and feasibility of PAMCAI's efficacy and cost‐effectiveness for evaluation in a larger randomised controlled trial (RCT). Using a two‐arm, single‐blinded pilot RCT with cost‐utility analysis, PAMCAI was compared with usual care in a tertiary hospital occupational therapy outpatient clinic. The primary outcome was adherence to wearing compression stockings, measured on a 4‐point scale. Twenty participants were randomised to receive PAMCAI (*n* = 10) or usual care (*n* = 10). Recruitment and pilot methodology appear feasible for use in a larger study. Compression stocking adherence was greater with PAMCAI compared to usual care (*p* = 0.002) and PAMCAI is likely cost‐effective, with an incremental cost‐effectiveness ratio of $3379.36 per quality‐adjusted life year (QALY) gained. Resolution of identified barriers to stocking adherence was positively associated with improvements in patient adherence. These findings support further investigation of PAMCAI in a larger RCT and its potential to improve patient adherence and the cost‐effectiveness of compression therapy for venous leg ulcer prevention.

**Summary**

- Using PAMCAI to identify and systematically address barriers to stocking adherence shows promise in improving patient adherence in a cost‐effective way, and a definitive RCT using the methods described in this paper is feasible.
- In this pilot study, adherence measured using a 4‐point scale significantly improved in the intervention group compared to the usual care group (*p* = 0.002).
- Resolution of individual patient barriers and improved stocking adherence appear to be linked.

## 1 Introduction

Venous leg ulcers (VLUs) are open wounds in the skin of the lower leg, most often seen in older adults, with underlying chronic venous insufficiency (CVI) [1]. The incidence varies between 0.73 to 3.12 cases per 1000 people annually [2], though variability across studies is common [3]. VLUs impose a significant financial burden on healthcare systems, with annual costs in Australia alone estimated at AUS$3 billion [4], while negatively impacting the quality of life for millions of individuals globally [1, 5, 6]. Medically prescribed compression stockings are the primary treatment for both healing and preventing VLUs [1, 5]. Consistent use, usually all day, every day, can hasten healing, reduce recurrence by over 50%, and improve quality of life [1, 7, 8]. However, worldwide adherence rates are low, ranging from 12% to 52% [1, 9], which is problematic since adherence is required to gain health benefits [10, 11], and to reduce the financial burden that VLUs impose upon healthcare systems and society [2].

It has been proposed that improving stocking adherence may offer greater benefits for patients than advancements in wound or medical treatments [12]. However, efforts to improve adherence have so far shown limited success [13]. Given that barriers to adherence are often patient‐specific and varied, multidimensional and tailored interventions are likely to be the most effective approach [13, 14]. To address this, the ‘Personalised and Multi‐dimensional Compression Assessment and Intervention’ (PAMCAI) was developed, encompassing a questionnaire, a clinical decision aid, and tailored strategies to improve adherence [14]. To date, neither the efficacy nor cost‐effectiveness of PAMCAI or other multidimensional interventions has been robustly investigated through RCTs or with larger cohorts. The aim of this pilot feasibility study was to assess the methods and feasibility, including clinical and cost‐effectiveness, of studying PAMCAI within a definitive RCT.

## 2 Methods

### 2.1 Study Design

A two‐arm, single‐blinded pilot RCT, including a within‐trial health economic evaluation (from the health service perspective with a 6‐month time horizon), was conducted within two occupational therapy vascular outpatient clinics within a tertiary hospital on the Gold Coast, Australia. Reporting followed the CONSORT checklist for pilot and feasibility trials [15]. Data on the impact of PAMCAI on clinician behaviour was also collected and will be reported separately. The research is registered with the Australian New Zealand Clinical Trials Registry (ANZCTR): 12620000544976. Ethical approval was granted through the Gold Coast Hospital and Health Service Human Research Ethics Committee and Bond University Human Research Ethics Committee: HREC/2020/QGC/62843 and SSA/2020/QGC/62843.

### 2.2 Participants and Recruitment

Potential participants included patients aged 18 and over who were re‐presenting or newly referred to the occupational therapy vascular service and were willing and able to give informed consent. Initial eligibility screening, conducted by the principal investigator (PI), was based on references to past VLUs in referrals or clinical documentation. An additional criterion was the possession of an Australian Commonwealth government concession card to ensure equal access to compression stockings for all research participants. Eligible concession cards [16] included the Pensioner Concession Card (for pensioners or certain social security recipients under specific conditions), the Disability Support Pension (for those with a long‐term diagnosis) or the Health Care Card (for social security and low paid workers). Patients meeting the eligibility criteria were contacted by the PI by phone to inform them about the study. To be included, patients needed to score above a threshold score of 3 on the Adhesig Screen [17], which was completed during the call. The Adhesig Screen grades compression stocking adherence using five questions (scored from 0 to 4) and is recommended for use as a screening tool with a sensitivity of 88.1% and specificity of 63.1% [17]. It was selected for its practicality in identifying potential candidates quickly. Eligible patients were then sent consent information along with a self‐addressed return envelope, and signed consent was received either by mail or email.

### 2.3 Clinicians

All participants attended their initial and final appointments with the usual clinicians within the service. All clinicians were occupational therapists at Queensland Health Practitioner Levels 3 or 4 [18], which are equivalent to entry level or senior clinician respectively. Their experience in the vascular specialty ranged from 4 months to 13 years, with an average of 2.5 years.

### 2.4 Groups

#### 2.4.1 Usual Care

Participants in the usual care group received routine occupational therapist care within the hospital outpatient service. This included (a) assessment (monitoring leg circumference at standardised sites, evaluating skin integrity, and checking stocking sizing, appropriateness, and condition), (b) compression stocking prescription (typically providing two stockings per affected limb for 6 months of wear), and (c) education on stocking use, skin care, exercise, and leg elevation, as recommended by the therapist. A follow‐up appointment was typically scheduled for 6 months, and participants were informed that they could request an earlier appointment if needed. Any interaction in relation to barriers or adherence to stocking wear was at the discretion of the occupational therapist, as happens in usual care.

#### 2.4.2 Intervention

Participants in the intervention group received care from occupational therapists using PAMCAI in a face‐to‐face setting. PAMCAI is a multidimensional tool delivered via an iPad application (app).

An iPad was selected as the delivery method for this pilot study, based on documented benefits of portable, point‐of‐care tools in supporting clinical‐decision making and enhancing patient outcomes [19, 20]. PAMCAI guides clinicians through the following chronological resources to improve compression stocking adherence:

- An assessment questionnaire identifies the patients' top three barriers.

The assessment, called the ‘Barriers to Compression Questionnaire,’ [14] is an ‘information‐gathering’ tool with 24 questions to identify patient‐specific barriers to wearing stockings. Clinicians complete the questionnaire with the patient, rating each barrier on a Likert scale from 1 (mild) to 5 (severe), based on its influence on the patient not wearing the garment.

b A clinical decision aid (algorithm) within PAMCAI guides decision‐making (linking steps a) and (c)

Responses to the questionnaire are processed by an algorithm that identifies and displays the top three weighted barriers without ranking them. This visual representation of the barriers' relative importance serves as the first step in the clinical decision aid for the clinician. If more than three barriers have equal weight, the clinician uses their discretion to prioritise them.

c Resources are recommended to guide the development of a treatment plan tailored to the identified barriers

PAMCAI generates a list of recommendations and resources to address each identified barrier, including information sheets, video links, and clinical considerations tailored to each barrier. The clinician then uses these resources to develop a personalised treatment plan. Further details regarding the development of PAMCAI resources have been previously published [14], and are listed in Supporting Information S1.

### 2.5 Outcome Measures

Outcomes were divided into two categories: feasibility and clinical outcomes. Data were collected at recruitment, during the initial clinical appointment, and at 6 months. At recruitment, the PI gathered demographic and health metrics, along with recruitment data. Quality of life outcomes were assessed by the PI during a phone call before the first clinical appointment and again after the final clinical appointment.

#### 2.5.1 Feasibility Outcomes

- Study processes

The PI recorded recruitment rates, reasons for non‐recruitment and drop‐outs, and lost to follow‐up, including the number of patients screened, contacted by phone, and consented. Clinicians were asked to report any concerns about the usability of the app to the PI, which was documented in a designated spreadsheet.

#### 2.5.2 Clinical Outcomes

##### 2.5.2.1 Primary Clinical Outcome

The primary outcome of interest was patient adherence with compression stockings. In the absence of a gold standard tool for the measurement of patient adherence in relation to compression stockings [13], adherence was measured using Question 10 (Q10) from the venous clinical severity score (VCSS) [21, 22]. The VCSS is commonly used in the CVI population and is described below, under secondary outcome measures. VCSS Q10 measures ‘compliance’ with compression therapy, with a higher score representing higher compliance over the preceding 4 weeks: 0 = None, 1 = Intermittent, 2 = Most Days, 3 = Fully Comply [21]. Treating clinicians used this scale to score Q10. Challenges and limitations in relation to the application of the VCSS Q10 have been previously reported [23, 24]. Therefore, a modified scale with quantifiable thresholds was developed by the research team to clarify these categories, support greater inter‐rater reliability, and to align with the common practise of reporting adherence rates as percentages [25]. Modifying validated tools is occasionally practised, especially in pilot studies, to evaluate proposed data analysis methods [26], and to enhance the sensitivity of the studied phenomenon [27]. This modification quantified categories based on the estimated average percentage of waking hours that compression was worn (modified Q10): 0 = 1%–10%, 1 = 11%–50%, 2 = 51%–90%, and 3 = 91%–100%. An independent assessor, approved by the research team and supported by the PI, independently calculated these percentage scores using participant estimates of their hours of wear. Data were collected from the intervention group by clinicians using PAMCAI and from the usual care group by an assistant (post consultation). The following parameters aided this calculation: (a) If stockings were worn on both legs for different durations, scores were calculated separately for each leg and averaged for an overall percentage; (b) when multiple ranges (hours awake and hours stockings were worn) were recorded, the widest range was used; (c) for ranges of days per week, the lowest number of days was recorded. The percentage of waking hours that compression stockings were worn was calculated by dividing the estimated hours per day by the estimated hours awake, multiplying by the estimated days per week, dividing by seven, and then multiplying by 100. Both the original Q10 scores and the modified Q10 scores were analysed and presented for the primary outcome of adherence.

##### 2.5.2.2 Secondary Clinical Outcomes

- Demographic and baseline health information

Patient demographic details such as age, gender, education level, marital status, and smoking status were obtained from clinical documentation and phone interviews. Health information, including weight, height, number and severity of ulcers, and related hospitalizations or hospital outpatient care for VLU before and during the study period, was also collected.

- Average daily hours in stockings

The percentage of average daily hours in stockings used for the primary outcome measure calculation was treated as a distinct outcome measure.

- Venous clinical symptoms: Venous clinical severity score (VCSS) [21]

The VCSS is widely used to assess the severity of chronic venous disease, with strong reliability (coefficient of 0.6) [23, 28]. It was selected for this research as it was already utilised in occupational therapy vascular clinics, with clinicians being familiar with its clinical descriptors and scoring categories. The VCSS comprises 10 scores (summed scores ranging from 0 to 30), evaluating nine aspects of venous disease, including pain, varicose veins, oedema, skin changes, and VLU's [28], each scored from 0 to 3, with higher scores representing greater severity of symptoms. However, for Q10 of the VCSS, a higher score reflects higher stocking compliance. This reverse scoring implies that higher compliance suggests more disease, as higher compression is presumed to be needed. The 2013 revision of the VCSS acknowledges that the use of compression therapy typically reduces symptoms of CVI, and controversy remains about this clinical indicator [24]. In this pilot, the VCSS was scored as intended by developers and reported as such.

- Health‐related quality of life

The chronic venous insufficiency quality of life questionnaire (CIVIQ‐14) [29], consists of 14 questions, designed to assess three key dimensions of quality of life in patients with CVI: pain, physical, and psychological. The questionnaire demonstrates validity and internal consistency [30]. Each question is scored from 1 (no problem) to 5 (severe problem), with total scores ranging from 0 to 100. Higher scores indicate a poorer quality of life due to CVI [31].

The EuroQol 5‐dimension 5‐level (EQ‐5D‐5L) [32] is a generic quality of life tool for measuring overall health‐related quality of life by describing and valuing health states to generate a health state profile. It is known for its validity and responsiveness in detecting changes in health status [33, 34]. Health states are assigned a summary index score based on societal preference weights, referred to as ‘utilities,’ ranging from 0 (death) to 1 (perfect health) which are used to calculate quality‐adjusted life years (QALYs). The EQ‐5D‐5L includes a visual analogue scale (VAS) score, where individuals rate their overall health on a scale from 0 (worse imaginable health) to 100 (best imaginable health), with higher scores indicating better perceived health [35]. Despite its generic nature, it has been utilised in multiple studies involving patients with chronic venous insufficiency [36].

- The extent to which participant barriers to compression stocking adherence were identified and resolved

To identify barriers to adherence, clinicians assessing participants in the intervention group used the iPad app ‘Barriers to Compression Questionnaire,’ during the appointment. For usual care participants, the assistant used a paper‐based version of the questionnaire after the clinician appointment (so that clinicians were blinded to usual care participants). The PI assigned a pre‐determined point value (1 to 4) to each barrier based on whether it was addressed and resolved (i.e., did not reappear at 6 months). The four‐point scale follows: 1 point—Barrier not addressed and not resolved, 2 points—Barrier not addressed and resolved, 3 points—Barrier addressed and not resolved (an attempt was made), 4 points—Barrier addressed and resolved. To adjust for participants with fewer than three barriers, mean scores were used to generate a Mean Barrier Resolution Score for each participant.

- Costs

Service delivery costs for PAMCAI and usual care were derived from the 2023 Pricing Framework for Australian Public Hospital Services, Tier 2 outpatient services [37]. Resource usage, taken from the medical records, included details on the number and type of appointments in occupational therapy vascular clinics, VLU‐related hospital admissions or VLU‐related vascular surgical clinic appointments, and the provision of compression stockings and applicators. Compression stocking postage costs were also recorded by the PI.

### 2.6 Randomisation, Blinding, and Study Procedures

Participants were randomly assigned to either the intervention or usual‐care group via a computer‐generated randomisation schedule [38], conducted by a hospital clinician not directly involved in the research, with assignment concealed from both participants and treating clinicians. Complete blinding of treating clinicians was not feasible due to the use of an iPad for the PAMCAI application in the intervention group. However, clinicians in the usual‐care group were unaware of participants' inclusion in the study. For the intervention group, the treating clinician administered the Barriers to Compression questionnaire and the VCSS.

To maintain blinding for the usual‐care group, a therapy assistant collected data on participant barriers and compression wearing times by phone, 1 to 2 days post‐consultation, using a standardised script. All participants were scheduled for an initial appointment shortly after recruitment and a six‐month review, marking the completion of their intervention period. To ensure equitable access to care, stocking applicators (where needed) were covered by research funding. Occupational therapists and assistants received training from the PI, including practise with PAMCAI through two case studies on the iPad app. An optional refresher session was offered prior to their first use of PAMCAI. Data entry and maintenance of the research database were managed by the PI.

### 2.7 Sample Size

A pilot sample size calculation was based on the assumption that a meaningful change in stocking adherence would be a 1‐point change on the VCSS Compression Therapy four‐point interval subscore (Q10), (standard deviation 1.14, power of 0.8, alpha 0.05) [39]. This calculation indicated a need for 44 participants for a full RCT. Accounting for a 20% dropout rate, this was increased to 56. A pilot sample size of 20 was utilised, deemed sufficient for preliminary analysis [15].

## 3 Analyses

All analyses were performed using IBMM SPSS version 29.0.1.0 and Microsoft Excel, with statistical significance set at < 0.05. Data analysis followed intention‐to‐treat criteria [40]. Recruitment, retention, baseline demographic, and health information were presented descriptively. *p*‐values for between‐group baseline characteristics were not reported [41]. The Shapiro–Wilk test assessed the non‐normality of the primary outcome measure. The Mann–Whitney U test evaluated group differences in the primary outcome of adherence, measured by both the VCSS Q10 and the modified VCSS Q10. Secondary outcomes including the VCSS [23], average daily hours in stockings, CIVIQ‐14 [42], EQ‐5D‐5L utility, and VAS scores [43], were analysed using univariate regression. Adherence barriers for both groups were presented descriptively. Somers' D assessed the direction and strength of the association between the Mean Barrier Resolution Score and modified VCSS Q10 change scores. The costs for occupational therapy vascular outpatient clinic appointments were AUS$193 for face‐to‐face and AUS$177 for phone or telehealth [37]. Specialist vascular services and hospital admission costs are not described as no such costs were incurred during the study period. The cost of stockings and applicators, ranging from AUS$19.99 to AUS$891.92, was sourced from supplier catalogues and the hospital purchasing department.

- Health economic evaluation

A within‐trial cost‐utility analysis (CUA) [44] was undertaken from the health service perspective, using Quality Adjusted Life Years (QALYs) derived from EQ‐5D‐5L utility values, based on Australian population normative data [44], and plotted over time. Six‐month scores were extrapolated to 1 year, and the area under the curve was calculated. The incremental cost utility ratio (ICER) was calculated by dividing the difference in costs between the intervention and usual care by the difference in QALYs (Cost‐utility = (Cost_i—Cost_c)/(QALY_i—QALY_c)). Baseline disparity in utility values was accounted for using univariate regression [45, 46].

## 4 Results

### 4.1 Feasibility Outcomes

**FIGURE 1:**
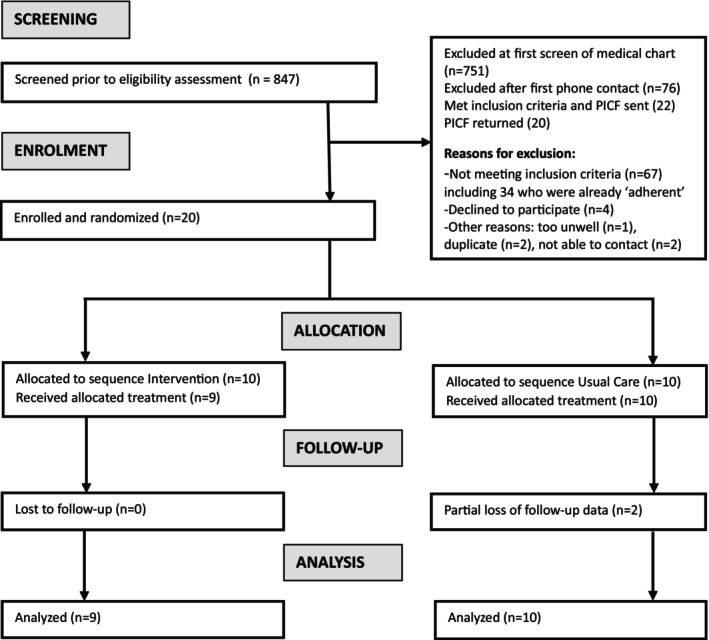
CONSORT flow diagram for recruitment.

From April 7 to October 1, 2022, a review of 846 referrals yielded 20 participants who were consented and randomised (Figure 1) [15]. Of these participants, eight were re‐presenting patients, three were from new referrals, and nine were from already scheduled appointments. One participant randomised to treatment withdrew prior to starting the intervention due to surgery for a comorbidity. Another participant was unreachable for the final study phone call; however, the analysis included earlier data from this participant. In the usual‐care group, one participant's final appointment was converted to a phone consultation, preventing the completion of the VCSS. Additionally, a clinician had to use a paper version of PAMCAI during one appointment due to a software update issue. Aside from this isolated issue, there were no reported usability problems with the iPad app that impacted patient care. Detailed usability findings will be presented in an upcoming qualitative paper. All clinicians invited to participate accepted without objections. While pilot recruitment was ultimately successful, delays were encountered due to the COVID pandemic and challenges associated with rotating staff schedules. During the study, several logistical hurdles emerged, notably with patient attendance at some appointments, often due to transportation difficulties and unforeseen medical events; however, none of these impacted the study outcomes.

### 4.2 Clinical Outcomes

#### 4.2.1 Demographic and Health Characteristics at Baseline

Baseline characteristics are presented in Table 1, which displays the means and standard deviations (where applicable) for each group's demographic and health metrics.

**TABLE 1:** Baseline demographic and health characteristics of randomised participants.

	Baseline characteristics	Usual care group (*n* = 10)	Intervention group (*n* = 10)
		Number (SD where applicable)	Number (SD where applicable)
Gender n (% women)		5 (50%)	4 (40%)
Age mean (SD) range		69.33 (17.41) 29–88	71.50 (11.36) 47–82
Education *n* (%)	Less than grade 10	2 (20%)	2 (20%)
	Grade 10 to grade 12	5 (50%)	2 (20%)
	Trade/certificate/diploma	2 (20%)	3 (30%)
	Undergrad/postgrad degree	1 (10%)	3 (30%)
Marital status *n* (%)	Single, divorced, separated, widowed	3 (30%)	6 (60%)
	Married, de facto	7 (70%)	4 (40%)
Smoker status *n* (%)	Never smoked	4 (40%)	2 (20%)
	Previous smoker	5 (50%)	6 (60%)
	Current smoker	1 (10.00%)	2 (20.00%)
BMI mean (SD)		28.84 (5.91)	36.79 (15.02)
VCSS Q10 mean (SD)		1.80 (1.13)	1.00 (0.86)^a^
Modified VCSS Q10 score mean (SD)		1.40 (0.30)	0.55 (24.00)^a^
Adherence Percentage score mean (SD)		52.09 (11.74)	19.51 (7.23)^a^
VCSS (scored by clinicians) mean (SD)		7.30 (2.90)	6.44 (1.94)^a^
CIVIQ‐14 GIS mean (SD)		38.00 (12.54)	39.00 (16.12)^a^
EQ‐5D‐5L—VAS mean (SD)		56.00 (20.92)	55.66 (30.75)^a^
EQ‐5D‐5L—utility score mean (SD)		0.70 (0.34)	0.59 (0.42)^a^

Abbreviations: BMI, body mass index; CIVIQ‐14 GIS, Chronic Venous Insufficiency Quality of Life Questionnaire‐14 Global Index Score; EQ‐5D‐5L, EuroQol 5‐Dimension 5‐Level Questionnaire, *n*, number of patients; VAS, Visual Analogue Scale; VCSS, Venous Clinical Severity Score.

^a^ *n* = 9 due to one participant withdrawing after randomization.

#### 4.2.2 Pilot Health Outcomes

- Primary outcome

**FIGURE 2:**
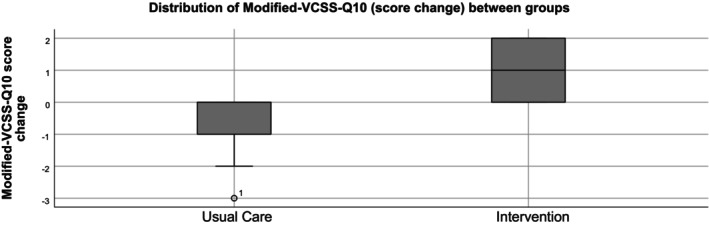
Distribution of post‐ minus pre‐study ‘Modified VCSS Q10 (score‐change)’ between groups.

Adherence scores improved significantly more in the intervention group compared to the usual care group for both the modified (*p* = 0.002) (Table 2 and Figure 2) and unmodified Q10 scores (*p* < 0.012) (Table 2). The modified VCSS Q10 scores were different from the unmodified scores in 50% of the total of 38 scores, equally distributed across groups (Supporting Information S2). In the usual care group, there was agreement between the modified and unmodified versions for 10 scores; 1 unmodified score was lower, and 9 were higher than the modified scores. Similarly, in the intervention group, 9 scores aligned; 1 unmodified score was lower, and 8 were higher.

- Secondary outcomes

**TABLE 2:** Primary and secondary outcome measures.

Outcome measure	Usual care	Intervention	Mean difference (intervention—usual care)	*p*
	(*n* = 10) mean (SD) change from baseline	(*n* = 9) mean (SD) change from baseline		
Primary outcome measure (Mann–Whitney *U*)				
VCSS Q10 mean change (SD)	−0.9 (1.28)	0.777 (1.20)	1.67	*p* = 0.012
Modified VCSS Q10 mean change (SD)	−0.60 (1.07)	1.00 (0.86)	1.60	*p* = 0.002
Secondary outcome measures (ANCOVA)				
Average daily hours in stockings (%) mean change (SD)	−24.82 (37.42)	38.574 (23.47)	63.40	*p* < 0.001
VCSS change mean (SD)	−0.40 (4.24)	0.94 (2.62)^b^	1.34	*p* = 0.552
CIVIQ‐14 GIS mean (SD)	−1.42 (35.39)^a^	1.23 (44.02)	2.65	*p* = 0.244
EQ‐5D‐5L utility scores mean (SD)	−0.054 (0.19)^a^	0.17 (0.30)	0.12	*p* = 0.156
EQ‐5D‐5L VAS scores mean (SD)	−7.22 (22.79)^a^	1.44 (45.25)	9.11	*p* = 0.344
A higher VCSS reflects worse venous symptoms, and a higher CIVIQ‐14 GIS indicates a poorer quality of life. For the other outcome measures above, a higher score indicates an improvement				
Barriers per group addressed and resolved (number and percentage)				
Total number of barriers per group: Usual care *n* = 28, intervention *n* = 25				
Barrier addressed and resolved (*n*) **%**	1 (3.57%)	18 (72.00%)	68.43%	
Barrier addressed and not resolved (*n*) **%**	7 (25.00%)	7 (28.00%)	3.00%	
Barrier not addressed and not resolved (*n*) **%**	20 (71.43%)	0 (0.00%)	−71.43%	
Barrier not addressed and resolved (*n*) **%**	0 (0%)	0 (0%)	0%	

Abbreviations: CIVIQ‐14 GIS, chronic venous insufficiency quality of life questionnaire‐14 global index score; EQ‐5D‐5L, EuroQol 5‐dimension 5‐level questionnaire; *n*, number of patients; VAS, visual analogue scale; VCSS, venous clinical severity score.

^a^ *n* = 9 due to one participant being unreachable for follow‐up phone assessment.

^b^ *n* = 8 due to one participant's final appointment being converted to a phone consultation.

**FIGURE 3:**
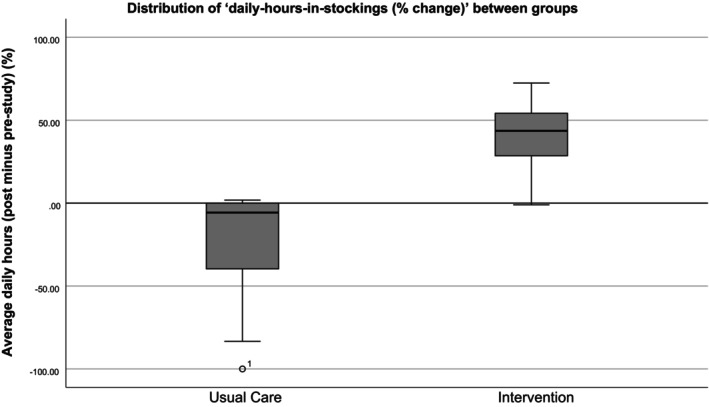
Distribution of post‐ minus pre‐study ‘daily hours in stockings (% change)’ between groups.

Average daily hours in stockings, expressed as a percentage, indicate that adherence improved significantly more in the intervention group than in the usual care group (*p* < 0.001) (Figure 3). Analysis of lower limb venous symptoms using VCSS revealed no significant difference between groups (*p* = 0.552). Similarly, the CIVIQ‐14 GIS scores showed no significant differences in quality of life between groups (*p* = 0.244). EQ‐5D‐5L Utility Scores demonstrated a slight improvement in the intervention group compared to a decrease in the usual care group, though these changes were not statistically significant (*p* = 0.156). Analysis of the EQ‐5D‐5L VAS scores indicated a decline in the usual care group and a modest improvement in the intervention group (*p* = 0.344). The intervention group resolved more barriers than the usual care group, presented in Tables 2 and 3. Table 3 also presents stocking adherence scores (VCSS modified Q10) alongside rates at which barriers were resolved (Mean Barrier Resolution Scores). Comparing Mean Barrier Resolution scores with their changes in modified VCSS Q10 yielded a Somer's D value of 0.471 (*p* < 0.001) signifying a significant positive association between barrier resolution and patient adherence with compression stockings. Sixteen of 24 possible barriers were selected by pilot participants. The most common were: ‘stockings were perceived as too hot to wear’ (usual care: 28.5%, intervention: 12.0%) and ‘difficulty donning stockings’ (usual care: 14.3%, intervention: 20.0%).

**TABLE 3:** Individual participant barriers, change in average daily wearing hours (%), change in modified VCSS Q10 scores, and mean barrier resolution scores.

Usual care										
	P01	P03	P05	P07	P10	P12	P14	P15	P17	P18
Top three barriers	Too hot	Too hot	Too hot	Can't don	Too tight	Too tight	Too hot	Too hot	Perception of looking old	Skin irritation
	Nil	Slides up foot	Skin irritation	Perception of looking old	Skin irritation	Too hot	Too tight over toes	Slides down	Can't don	Can't don
	Nil	Can't don	Perception of looking old	Digs in at ankle	Too hot	Skin irritation	Low mood	Digs in at top	Can't doff	Too hot
Change (%), modified Q10 score	−100% (−3)	−5.80 (0)	−5.61 (0)	−2.14% (0)	+1.81 (0)	−39.62 (−1)	−83.33 (−2)	−13.57% (0)	0% (0)	0% (0)
Mean barrier resolution score	1.00	1.00	1.67	2.33	2.00	1.67	1.67	1.00	2.33	1.00

Intervention										
	P02	P04	P06	P08	P09	P11	P13	P16	P19	P20
Top three barriers		Self‐efficacy	Digging in at ankle	Limited understanding why needed	Can't doff	Slides up foot	Can't don	Too hot	Too tight at base of toes	Skin Irritation
		Slipping down	Digging in at top	Can't don	Digs in at ankle	Low self‐efficacy	Low mood	Limited understanding why needed	Dressings and wounds	Can't don
		Nil	Can't don	Too hot	Low mood	Nil	Digging in at top	Low mood	Can't don	Too hot
Change (%), modified Q10 score		+28.57 (0)	+40.62 (+1)	+57.05 (+2)	+72.41 (+2)	+43.57 (+1)	+54.14 (+2)	−1.028 (0)	+44.089 (+1)	+7.718 (0)
Mean barrier resolution score		3.50	4.00	3.67	3.67	4.00	3.33	3.33	4.00	4.00

*Note:* 1 point—Red—Barrier not addressed and not resolved; 2 points—White—Barrier not addressed and resolved (nil noted); 3 points—Orange—Barrier addressed and not resolved (the clinician had attempted to address them); 4 points—Green—Barrier addressed and resolved.

#### 4.2.3 Pilot Economic Evaluation

Table 4 presents the appointments and costs by group. The total mean costs over 6 months were $1097.70 (SD $451.95) for the intervention group and $675.28 (SD $381.00) for the usual care group, indicating that the intervention is slightly more expensive than usual care. The difference in QALYs gained (adjusting for differences in baseline utility scores) [45, 46] was 0.125, indicating slightly greater improvement with the intervention. The base case ICER was therefore estimated at AUD$3379.36, suggesting cost‐effectiveness of PAMCAI when compared with a reference Australian willingness to pay ICER of AUD$28033 [47].

**TABLE 4:** Number of appointments, VLUs, VLU‐related hospital admissions and specialised VLU care, costs of care delivery, and cost utility analysis (ICER).

Variable	Usual care (*n* = 10)	Intervention (*n* = 9)	Mean difference (intervention—usual care)
	Number/cost (SD where applicable)	Number/cost (SD where applicable)	
Occupational therapy F‐2‐F Appointments: mean number	1.9	2.89	0.99
Occupational therapy phone appointments: mean number	0.5	1.22	0.72
Occupational therapy appointments: mean cost (SD)	$472.91, ($248.33)	$773.88, ($305.63)	$300.97
Specialised appointments related to VLU recurrence: mean number	0	0	0
Specialised appointments related to VLU recurrence: mean cost, (SD)	$0 ($0)	$0 ($0)	$0
Hospital admissions related to VLU recurrence: mean number	0	0	0
Hospital admissions related to VLU recurrence mean cost, (SD)	$0 ($0)	$0 ($0)	0
Materials: stockings, applicators, postage: mean cost, (SD)	$202.36, ($263.80)	$323.81, ($189.01)	$0
TOTAL: mean cost, (SD)	$675.28, ($381.01)	$1097.70, ($451.95)	$422.42
Cost utility analysis			
QALYS mean, (SD)	0.74, (0.24)	0.72, (0.33)	−0.02

*Note:* ICER of PAMCAI adjusted for baseline differences in EQ5D5L utility scores: $422.42/0.125 = $3379.36.

Abbreviations: EQ‐5D‐5L, EuroQol 5‐dimension 5‐level questionnaire; F‐2‐F, face‐to‐face; ICER, incremental cost effectiveness ratio; *n*, number of participants; VLU, venous leg ulcer.

## 5 Discussion

This is the first study to investigate a new multidimensional and personalised intervention (PAMCAI) aiming to improve stocking adherence. This pilot investigation indicates that PAMCAI resulted in significantly greater stocking adherence, and while slightly more expensive than usual care, was well below Australian reference ICER values, suggesting it is also cost‐effective. Furthermore, this is the first documented report of a positive association between the resolution of collaboratively identified barriers to stocking adherence (identified by use of the PAMCAI tool) with improvements in patient adherence to wearing compression stockings. Further investigation via a fully powered RCT is therefore warranted to investigate and validate these findings with a larger sample and across more diverse settings. This study also indicates that the methodology piloted is both acceptable to clinicians and feasible for implementation in a fully powered RCT.

Unexpectedly, this pilot demonstrated a significant result for the primary outcome as, despite low numbers, a large effect size was observed. This was calculated by using the modified VCSS Q10 score, maintaining the assumption that a 1‐point change on this scale is meaningful and using a two‐tailed Mann–Whitney U test in G*Power [46], applying the asymptotic relative efficiency (ARE) method with alpha set at 0.05 and power set at 0.8. This resulted in a calculated effect size for the pilot RCT of 1.64, indicating that 18 participants are required for a definitive trial. While this pilot study included only 19 participants, the large effect size showed significant results, suggesting that the small sample size was adequate to inform on the primary outcome measure of adherence. However, to explore additional outcome measures, perform multivariable regression analysis, and to compensate for potential dropouts, a larger sample size will be necessary.

As anticipated in the pilot with a short time horizon, PAMCAI resulted in only slightly better QALYs compared to usual care and similarly, mean CIVIQ‐14 GIS scores were only marginally higher, without reaching statistical significance. These findings might be attributed to the confounding effects of comorbidities, especially prevalent in the cohort under investigation [48]. It may also be related to the study's short duration, since meaningful improvements in quality of life might require consistent adherence over an extended period, potentially spanning years, especially when an intervention is multidimensional compared to simpler interventions [49]. These findings support the need for a longer follow‐up period, such as 2 years or longer, in a larger RCT. Similarly, data on VLUs and related care were collected, noting the absence of VLU recurrences during the study period and this can also be addressed with a longer follow‐up in a subsequent larger RCT. Additionally, while two participants received care for leg wounds (trauma and cancer treatment) that did not meet VLU criteria during the study, these wounds were likely influenced by pre‐existing CVI, which may have affected the study findings [50]. Managing these challenges in a fully powered RCT may involve expanding inclusion criteria to include patients with earlier CVI stages [9], including oedema and skin changes, alongside those with a history of VLU. Ensuring an adequate sample size may also allow randomisation to sufficiently balance confounding variables across treatment groups.

Adding further nuance to interpretation, the level of baseline adherence among participants, such as partial adherence, might already have conferred some benefits. For instance, in patients with chronic conditions such as osteoporosis, who are already partially compliant with recommendations for treatment, further enhancements in adherence often result in incremental quality of life benefits, rather than large gains [51].

The observed positive association between the resolution of patient‐specific barriers and improved adherence is a new finding that has not previously been reported in the compression stocking literature. It highlights the potential importance of tailoring interventions to individual patient circumstances and prompts reconsideration of a one‐size‐fits‐all approach to adherence challenges [52]. The likely cost‐effectiveness of PAMCAI suggests that its slightly higher costs from additional appointments are justified by the personalization of care, where additional time allows clinicians to better address individual barriers. PAMCAI aligns with recommendations for implementing personalised care in healthcare and embodies the principles of ‘P4 Medicine’ [53] (predictive, preventative, personalised and participatory). This approach, which focuses on being proactive rather than reactive, wellbeing rather than disease, individual‐oriented in addition to population‐oriented, has the potential to improve the quality of clinical care while ultimately reducing costs [54]. It follows that PAMCAI is predictive, as it anticipates which individual patient barriers are likely to interfere with adherence. It is preventative in nature, as it provides resources for clinicians to improve adherence, such as providing alternative methods of stocking application to prevent symptom progression. It is personalised, as the assessment identifies patient‐unique barriers, tailoring solutions to their unique circumstances. Finally, it is participatory, actively involving patients in identifying their barriers and collaborating in the development of their treatment plan to find effective solutions.

Future integration of PAMCAI into the care pathway requires the development of training packages, support for administrative systems integration, and further evaluation of implementation resources. These components have the potential to be explored in a future RCT. Additionally, future iterations could include tailored versions of PAMCAI for children and individuals with lymphedema, broadening its scope and potential impact.

A strength of this study lies in the extensive clinical experience of the clinicians involved in its design. This ensured a deep understanding of real‐world adherence challenges and nuances, thereby enhancing the relevance of research outcomes in clinical practise. Another strength of the study is its robust research methodology, conducted within a tertiary hospital. The study benefited from rigorous oversight by the hospital's research department, in collaboration with a university, leveraging the expertise of experts from diverse fields.

Some limitations are acknowledged. Firstly, a selection bias may be present due to the requirement for participants to provide consent, potentially excluding those not attending outpatient clinics. Secondly, the reliance on self‐reported data introduces the risk of recall bias or a desire to please the clinician [55]. While a four‐week recall period was used to align with the CIVIQ‐14 [29], a shorter period may have gained more accurate responses [56]. Diaries were considered, but may also be compromised by a social desirability, exaggeration, under‐reporting, and poor memory [57]. Objective methods, such as wearable technology [58, 59] offer alternatives. While these methods can be costly and may not perform reliably in all climates [17], they merit consideration for future research as technology advances. Thirdly, in the absence of a gold standard outcome measure for adherence [13], the VCSS was an appropriate tool; however, there are limitations, particularly with the inverse scoring of question 10 and its influence on the overall venous symptoms score [24, 60]. We considered summing only the first nine questions; however, this option was discarded because the assessment is validated to include all 10 questions. The VCSS Q10 modification and a scaled scoring for barrier resolution aimed to enhance the level of detail to inform our research objectives. Both the modified and unmodified adherence scores demonstrated significant findings (*p* = 0.002 and *p* < 0.012), but the modified score may have captured more detailed variability in adherence and addressed a potential bias identified through score comparisons (Supporting Information S2). Specifically, clinicians' scoring of adherence was more conservative (lower) when using the modified scale in comparison to the original unmodified version. This difference suggests that further evaluation of the modification and research to enhance measures of stocking adherence are warranted.

The percentage calculation performed to allocate an adherence score (time in stockings ÷ time awake x 100) may benefit from further exploration. The calculation appears to offer a practical and objective measure that is sensitive to change. Incorporating these percentage‐based nuances into the validated VCSS could enhance the VCSS's utility in future research on stocking adherence. Alternatively, using this percentage‐based method independently of the VCSS could be a practical approach, as it does not require specialised assessment forms or training. Clinicians can collect data by asking straight‐forward questions about their sleep and wake times, as well as when they put on and take off their stockings, to calculate adherence percentages. Of note, clinicians sometimes recorded participants' awake and stocking wearing times as ranges, necessitating the development of post‐data collection scoring rules. Calculating the mid‐point of the reported time range might have been a more appropriate approach.

Finally, the pilot study's generalizability may be limited by its single‐site setting on the Gold Coast, Australia, where the warm, humid climate, resources, and expertise of a major teaching hospital could influence adherence and patient outcomes differently than in smaller or less‐equipped facilities. During the resource development phase [14], efforts were made to ensure the recommendations in PAMCAI considered universal barriers and strategies. Additionally, PAMCAI is designed for use independent of hospital systems, such as solo therapists working in private practise or isolated posts where clinical supervision may be limited. Further evaluation in a multi‐site RCT would aid the generalizability of research outcomes.

## 6 Conclusions

A pilot RCT testing PAMCAI, which aimed to improve patient adherence to compression stockings, demonstrated the feasibility of the research methodology and identified key recommendations for recruitment, outcome measures, and trial design for a definitive RCT. Despite the small sample size, the pilot showed statistically significant improvements in stocking adherence due to a large effect size, likely cost effectiveness, and demonstrated a positive association between the resolution of patient barriers and improved stocking adherence. These findings indicate that PAMCAI shows promise as a new tool to improve compression stocking adherence and that further research with a larger sample and in multiple settings is warranted.

## Conflicts of Interest

The authors declare no conflicts of interest.

## Supporting information


**Supporting Information S1.** List of PAMCAI's Resources (the tailored strategies).

**Supporting Information S2.** Comparison between clinicians' scores and the modified scores for VCSS question 10.

## Data Availability

Data available on request from the authors.
