# Supplementary material for: Adherence to Compression Stockings for Venous Leg Ulcer Prevention: A Pilot Randomised Controlled Trial and Health Economic Analysis, Evaluating a New Multidimensional Tool (PAMCAI)
Source: Int Wound J. 2025 Apr 15;22(4):e70244. doi: 10.1111/iwj.70244 (PMC11999730; doi:10.1111/iwj.70244)
Supplement: Supplementary file 1 — Supporting Information S1. List of PAMCAI's Resources (the tailored strategies). [file IWJ-22-e70244-s002.docx]

Supplementary file 1. List of PAMCAI’s Resources (the tailored strategies)

| **Type of resource** | **Title** |
| --- | --- |
| Handouts: Applicators | Ezy-As – carers version |
|  | Ezy-As – patient version |
|  | Heel-out method – carers version |
|  | Heel-out method – patient version |
|  | Jobst Metal Frame – carers version |
|  | Jobst Metal Frame – patient version |
|  | Sally Stocking Aid – carers version |
|  | Sally Stocking Aid – patient version |
|  | Sigvaris Doff N Donner – carers version |
|  | Sigvaris Doff N Donner – patient version |
|  | Silk Foot Slippee – carers version |
|  | Silk Foot Slippee – patient version |
|  | Steve Plus Applicator – carers version |
|  | Steve Plus Applicator – patient version |
|  | Medi-Butler Off patient handout |
| Handouts: General information | Correct Garment Positioning |
|  | General Leg Care |
|  | How Do Stockings Work? |
|  | How To Care for your Stocking |
| Videos for patients/carers | Ezy-As – carers version |
|  | Ezy-As – patient version |
|  | Heel-out method – carers version |
|  | Heel-out method – patient version |
|  | Jobst Metal Frame – carers version |
|  | Jobst Metal Frame – patient version |
|  | Sally Stocking Aid – carers version |
|  | Sally Stocking Aid – patient version |
|  | Sigvaris Doff N Donner – carers version |
|  | Sigvaris Doff N Donner – patient version |
|  | Silk Foot Slippee – carers version |
|  | Silk Foot Slippee – patient version |
|  | Steve Plus Applicator – carers version |
|  | Steve Plus Applicator – patient version |
| Information sheets for clinicians | Common problems and how to fix them |
|  | Correct stocking position |
|  | How to care for stockings |
|  | How to measure for a stocking |
|  | Types of compression |
|  | Types of Medical Conditions requiring compression |
|  | When NOT to compression |
|  | Why clinician interactions are so important |
|  | Why do stockings work |
|  | Why self-efficacy is so important |
| Answer Sheets | 1. Are stockings digging in at the ankle? |
|  | 1. Do stockings feel too tight? |
|  | 1. Do stockings slide down the leg? |
|  | 1. Are stockings digging in at the top of the leg? |
|  | 1. Are stockings too tight at the base of the toes? |
|  | 1. Are stockings too tight over the toes? |
|  | 1. Is the fabric irritating the skin? |
|  | 1. Is the foot section sliding up the toes? |
|  | 1. Is there a large foot compared to a thin leg? |
|  | 1. Do stockings feel too hot to wear sometimes? |
|  | 1. Is there irritation from a silicone grip top |
|  | 1. Does the patient have limited understanding about their medical condition? |
|  | 1. Is there limited understanding about their medical condition? |
|  | 1. Was there a previous negative experience? |
|  | 1. Was there previous conflicting advice? |
|  | 1. Could the patient have low mood? |
|  | 1. Could the patient have low self-belief in their own abilities? |
|  | 1. Is there concern stockings will make them look old? |
|  | 1. Is the patient unable to put stockings on? |
|  | 1. Does the patient have wounds or dressings? |
|  | 1. Is the patient unable to get stockings off? |
|  | 1. Does the patient not know how to take care of stockings (knowledge)? |
|  | 1. Does the patient have difficulty taking care of stockings (memory/planning)? |
|  | 1. Does the patient not know who to contact if having problems or their stockings need replacing? |
| Other | Templates for documentation |
|  | Template for ‘barrier identification’ to development of a plan |
|  | Frequency of Wear recording form |
|  | Barriers to Compression Questionnaire paper form |
