# Supplementary material for: Adherence to Compression Stockings for Venous Leg Ulcer Prevention: A Pilot Randomised Controlled Trial and Health Economic Analysis, Evaluating a New Multidimensional Tool (PAMCAI)
Source: Int Wound J. 2025 Apr 15;22(4):e70244. doi: 10.1111/iwj.70244 (PMC11999730; doi:10.1111/iwj.70244)
Supplement: Supplementary file 2 — Supporting Information S2. Comparison between clinicians' scores and the modified scores for VCSS question 10. [file IWJ-22-e70244-s001.docx]

Supplementary file 2: Comparison between clinicians’ scores and the modified scores for VCSS question 10

| **Alignment** | **Usual Care (n=20)** | **Intervention (n= 18)** |
| --- | --- | --- |
| Clinician score aligned with modified score | 10 | 9 |
| Clinician overscored by 1 | 7 | 8 |
| Clinician overscored by 2 | 2 | 0 |
| Clinician underscored by 1 | 1 | 1 |
